# Circulars and Bulletins from the Office of the Chief Surgeon

**Published:** 1918-08

**Authors:** 


### CIRCULARS AND BULLETINS FROM THE OFFICE OF THE CHIEF SURGEON

Medical officers with the American Expeditionary Forces in France are not only interested in the individual work that they are doing, but they feel a pride in what has been accomplished, and in what is at present being done by the Medical Department of which they are a part. They have the greatest respect for, and confidence in, the ability and judgment of the Chief Surgeon and his Staff. They know that whatever is sent out from the Chief Surgeon's Office has been carefully considered by men of unusual ability and with long experience in military surgery; and that the circulars, bulletins, and reports that are issued from Medical Headquarters are not only an official expression of the policies which should guide the work of every medical officer in France, but that they represent the best medical thought on the subjects with which they deal.

Beginning with this issue, *War Medicine* will publish each month extracts from the official circulars and bulletins that are sent out from the office of the Chief Surgeon, and it is believed that this department will prove an addition of real value to those whom we desire to serve. These circulars and bulletins are sent to the commanding officers of hospitals, to all division surgeons, and to the heads of other medical units for distribution, but many medical officers do not receive them. Whether they receive them or not, every medical officer desires to have these important documents in a permanent form, and properly indexed, so that they may be referred to as the need for official advice is felt. This is accomplished by publishing them in *War Medicine*.

Of course, if any circular or bulletin should contain information that could be regarded as confidential, or of such nature that it would give aid to the enemy it would not be published. Likewise those which deal with small groups of men and which would not be of general interest will be omitted.

In this number are published extracts from recent circulars, sent out from the Chief Surgeon's Office, dealing with a number of matters pertaining to administration in the Medical Department of the A. E. F., in which all medical officers are interested; but special attention is called to the plan of organization of the professional services with the American Expeditionary Forces.

The Chief Surgeon makes no distinction between the Regular Medical Corps and the Medical Reserve Corps; but he has wisely placed the medical officers who have had long experience with military administration in executive positions, and has placed the care and treatment of the sick and wounded soldiers very largely in the hands of physicians and surgeons who have had large clinical experience. This circular outlines very definitely a plan for an organization in which the best clinical medical talent from America is utilized. It has been in operation for some time, and it is announced, because the Chief Surgeon knows that it makes for the highest degree of efficiency in safeguarding the health and lives of our soldiers.

The Bulletins from the Chief Surgeon's Office deal with disease. They always contain a great deal of interesting data, expressed in very attractive style; and much other important information that all medical officers should have. The notes in these Bulletins on infectious diseases, and preventive medicine generally, are intensely practical; and they place in the hands of medical officers first-hand facts regarding military hygiene and sanitation, as applied to conditions in the A. E. F., that should be helpful to them in their efforts to reduce the morbidity and mortality rates in our Army.

